# Supplementary material for: Delineating organizational principles of the endogenous L-A virus by cryo-EM and computational analysis of native cell extracts
Source: Commun Biol. 2024 May 10;7:557. doi: 10.1038/s42003-024-06204-7 (PMC11087493; doi:10.1038/s42003-024-06204-7)
Supplement: Supplementary file 2 — Supplementary Information [file 42003_2024_6204_MOESM2_ESM.pdf]

SUPPLEMENTARY MATERIAL

Delineating organizational principles of the endogenous L-A virus by cryo-EM and computational analysis of native cell extracts

Lisa Schmidt[1,2,#], Christian Tüting[1,2#*], Fotis L. Kyrilis[1,2,3], Farzad Hamdi[1], Dmitry A. Semchonok[1], Gerd Hause[4], Annette Meister[1,2], Christian Ihling[5], Milton T. Stubbs[1,2], Andrea Sinz[5], and Panagiotis L. Kastritis[1,2,3,5*]

[1]Interdisciplinary Research Center HALOmem, Charles Tanford Protein Center, Martin Luther University Halle-Wittenberg, Kurt-Mothes-Straße 3a, Halle/Saale, Germany.

[2]Institute of Biochemistry and Biotechnology, Martin Luther University Halle-Wittenberg, Kurt-Mothes-Straße 3, Halle/Saale, Germany.

[3]Institute of Chemical Biology, National Hellenic Research Foundation, Athens, Greece.

[4]Biozentrum, Martin Luther University Halle-Wittenberg, Weinbergweg 22, Halle/Saale, Germany.

[5]Institute of Pharmacy, Center for Structural Mass Spectrometry, Martin Luther University Halle-Wittenberg, Kurt-Mothes-Str. 3, 06120, Halle (Saale), Germany

[#]These authors contributed equally

* Correspondence to:

Jun.-Prof. Dr. Panagiotis L. Kastritis

Weinbergweg 22, 06120 Halle (Saale) / Germany

Mail: panagiotis.kastritis@bct.uni-halle.de

Dr. Christian Tüting

Weinbergweg 22, 06120 Halle (Saale) / Germany

Mail: christian.tueting@biochemtech.uni-halle.de

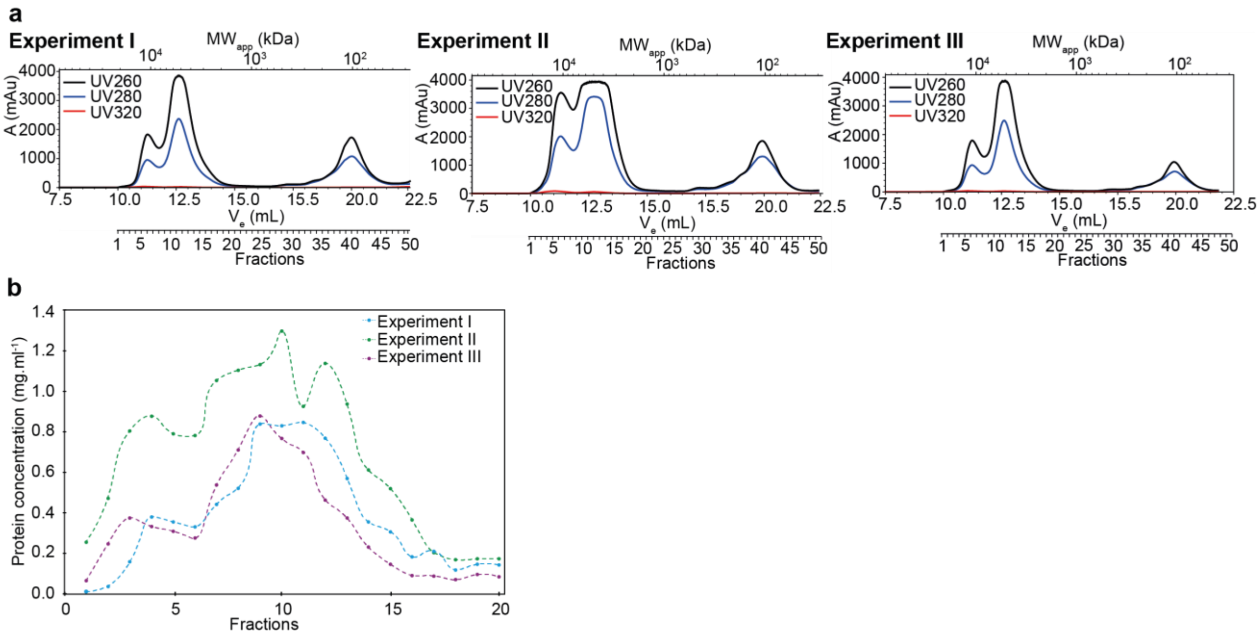

Supplementary Fig. 1 Identification of the L-A virus: (a) Triplicate SEC at 260 nm, 280 nm, and 320 nm absorbance. (b) Triplicate of the experiment showing high in-fraction protein concentration of the resulting fractions after SEC measured with Bradford reagent.

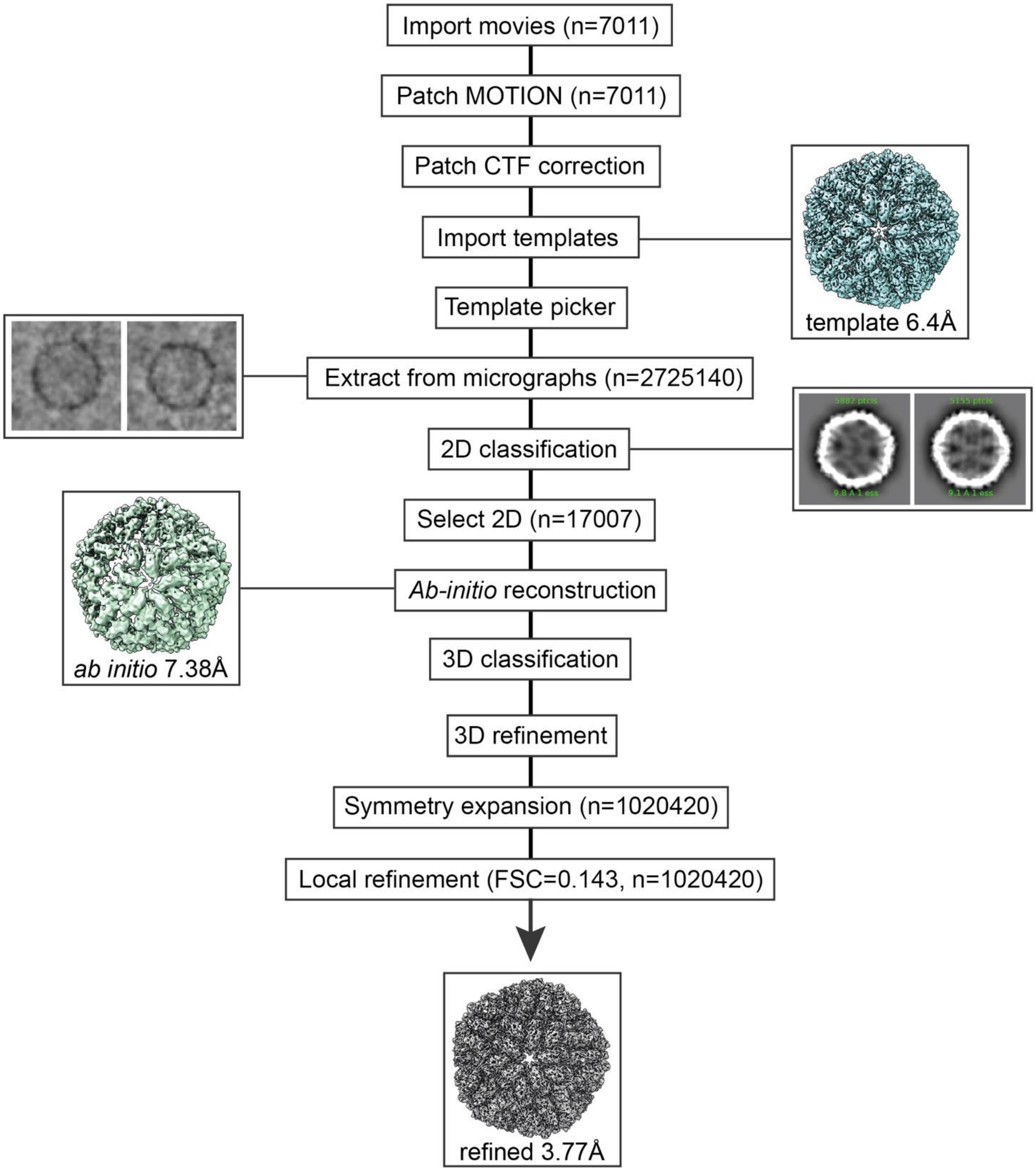

Supplementary Fig. 2 Workflow of image analysis: Shown is a workflow of image analysis, specifically followed for the high-resolution cryo-EM structure of the L-A virus. Data is imported into the workspace, followed by motion correction and CTF correction procedures. After these processes finished, particles can be picked, and a *de novo* lower resolution reconstruction is derived after 3D classification and refinement. For template picking this reconstruction was chosen. The picked particles are then extracted and 2D classified. Selected 2D class averages were used for another *ab initio* reconstruction, followed by 3D classification and 3D refinement of chosen particles. The map quality was improved after using symmetry expansion and particles were, lastly, locally refined.

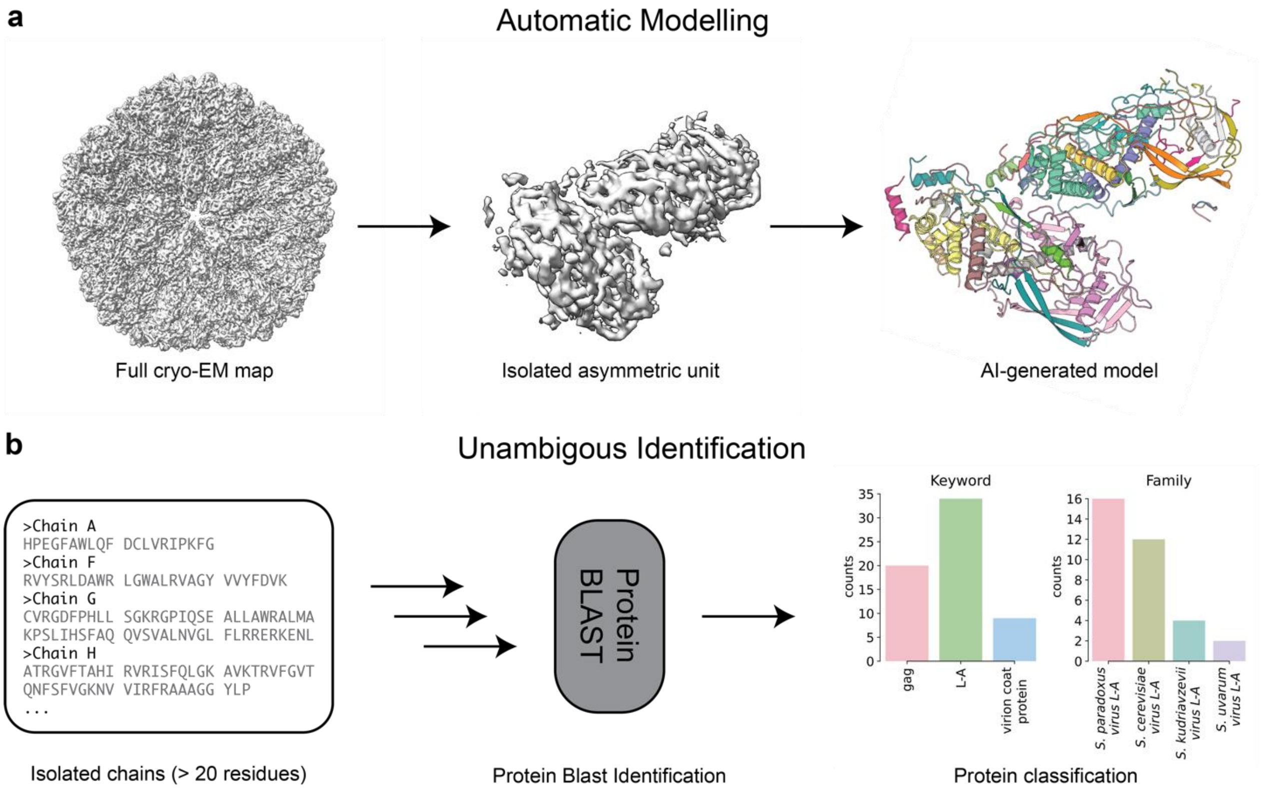

Supplementary Fig. 3. Unsupervised atomic modelling of the L-A virus 3.77 Å resolution capsid with ModelAngelo. (a) Automated modelling: From the cryo-EM map, the asymmetric unit was isolated. This extracted density was then served as input for ModelAngelo without incorporating any sequence information to produce an atomic model. The output model is notably fragmented and is visualized using a cartoon representation. Chain fragments are color-coded for clarity. (b) Unambiguous Identification: Fragments possessing a length of 20 residues or higher (*N*=19) served as input to the NCBI protein BLAST (BlastP). Following protein identification in NCBI, entry fields “keyword” and “family” were extracted and their absolute counts were displayed as bar plots. The most recurrent keywords observed included "gag," "L-A," and "virion coat protein." Concurrently, the dominant protein families identified comprised of L-A viruses in different yeasts, which also included S. cerevisiae among the top hits. In summary, given that our organism of interest was baker's yeast, the viral protein visualized in the cryo-EM data is associated with the *S. cerevisiae L-A* virus. Notably, this conclusion was drawn in a completely unsupervised and unbiased manner, showing the potential of unsupervised atomic modelling for protein identification.

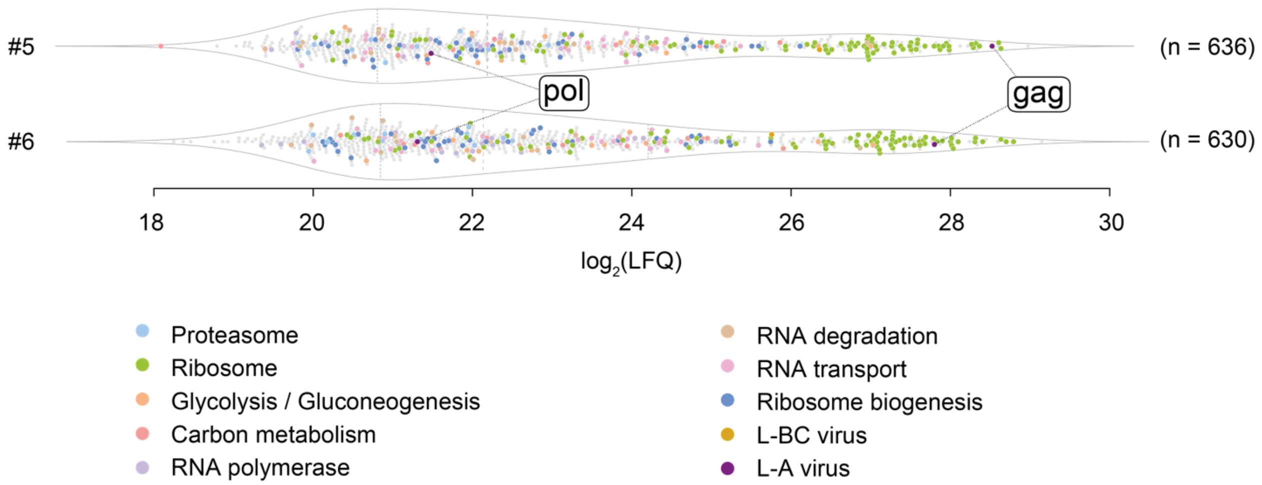

Supplementary Fig. 4 Mass spectrometry data for in-fraction protein content. Label-free quantification of the protein content of fractions 5 and 6 showing the virus proteins, translation-related proteins, proteins for glycolysis, carbon metabolism, spliceosome, citrate cycle, and others, present in the same cellular fraction. n defines the number of identified proteins. Technical duplicates of biological triplicates were analysed per fraction.

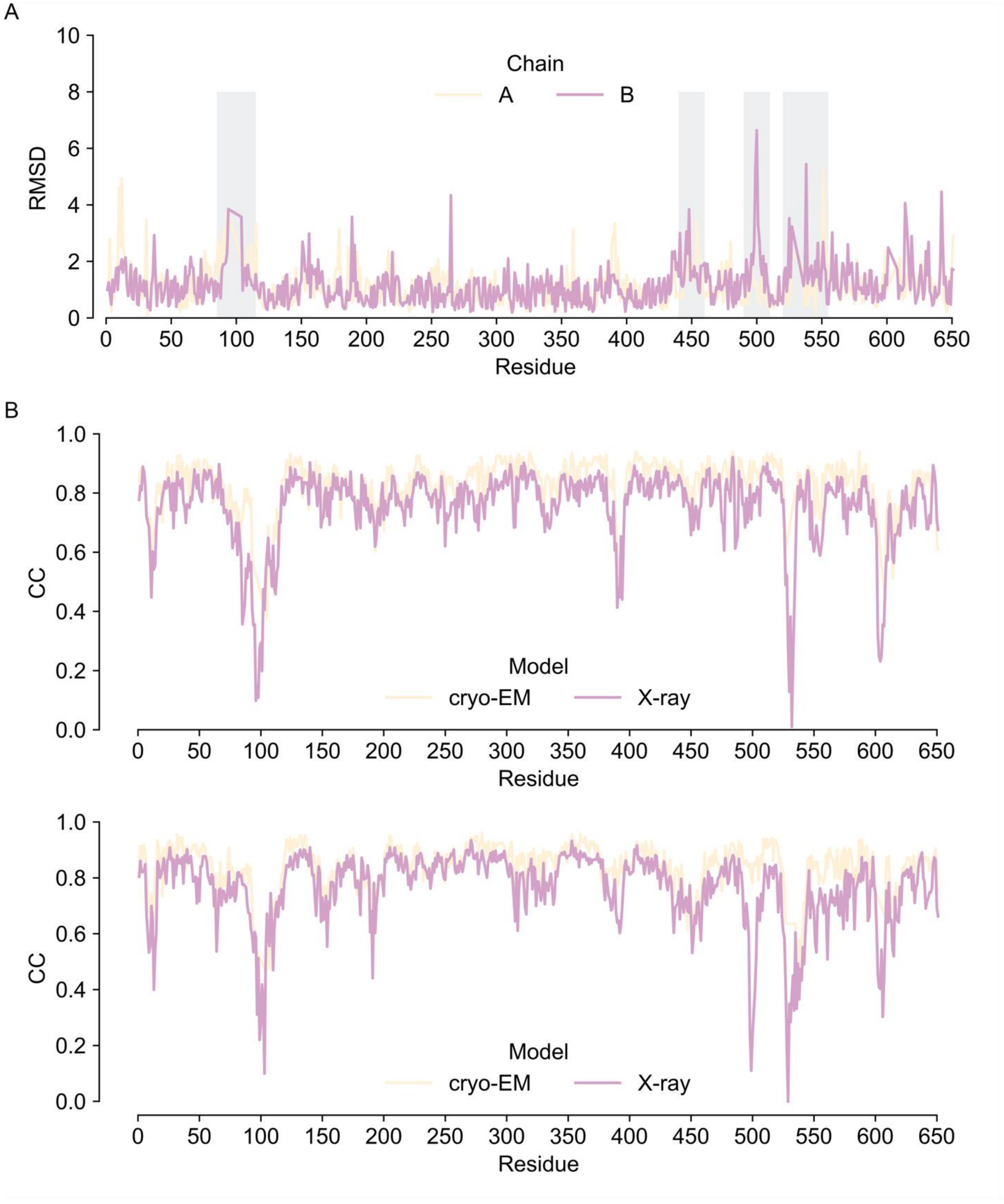

Supplementary Fig. 5. Fits and conformational variations of the X-ray and cryo-EM models to the resolved cryo-EM map: (a) Root Mean Square Deviation (RMSD, Å) between the cryo-EM and the X-ray structure per capsid protomer. In the depicted line graphs, *Protomer A* is represented by a yellow line and *Protomer B* by a purple line. Flexible regions discussed in the manuscript and shown in Fig. 3 are emphasized using a *grey box* overlay. (b) Cross-

correlation of the cryo-EM (peach) and the X-ray (purple) models of chain A and chain B are shown in this plot, indicating local conformational variability as dips in cross-correlation values.

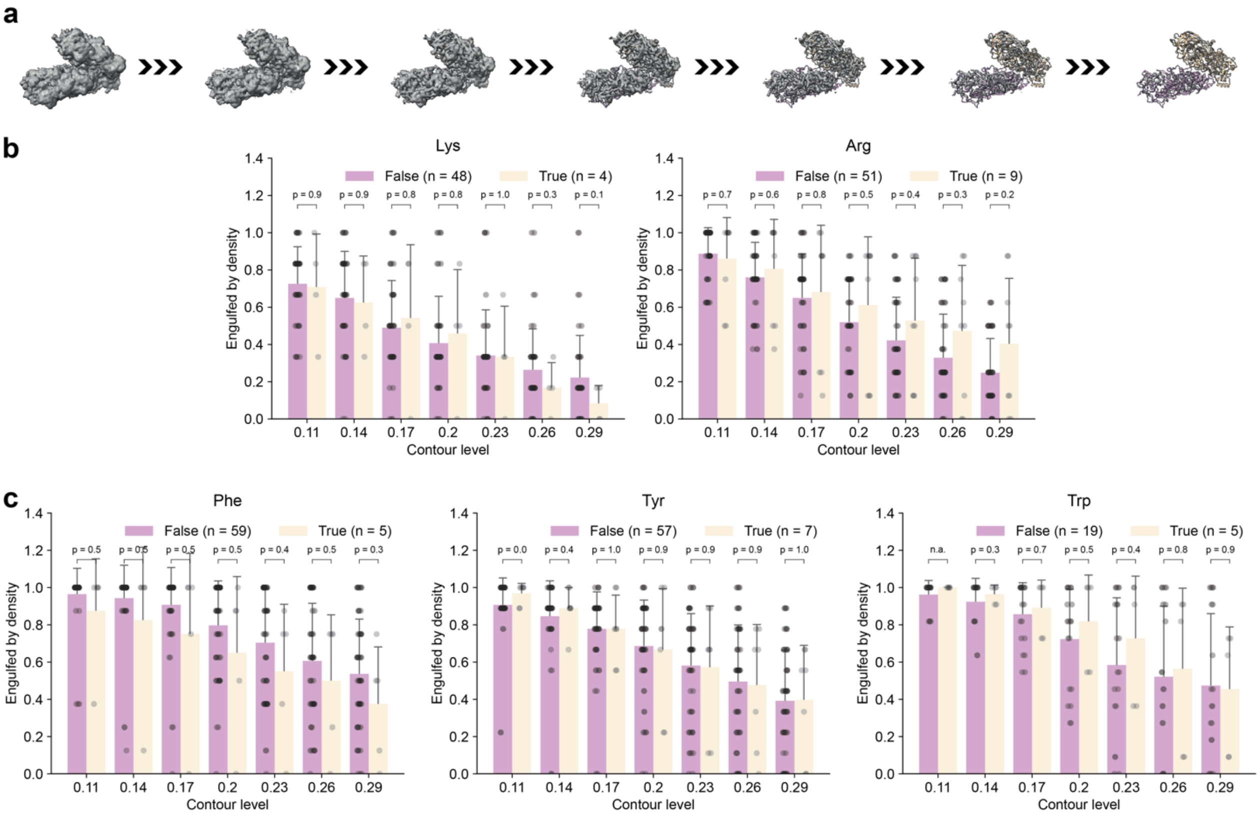

Supplementary Fig. 6. Analysis of cation-π interactions in the viral capsid. (a) A stepwise reduction of the density map highlights areas of high resolution, showcasing the per-atom engulfment calculations shown in (b) and (c). (b) Per-atom engulfment results for cationic and (c) aromatic residues from the cryo-EM model. The bar plots display engulfment values for each residue type: x axis shows the contour level and the y axis represents relative engulfment. Residues are differentiated based on their involvement in cation-π interactions, labeled as "True" or other interactions, labeled as "False". P-values, calculated using a two-sample t-test with unequal variances, are annotated where statistical comparison was feasible; elsewise, "n.a." notation is shown. The error bars indicate the sample standard deviation, the n values the number of identified residues in their free (False) or cation-π coordinated state (True).

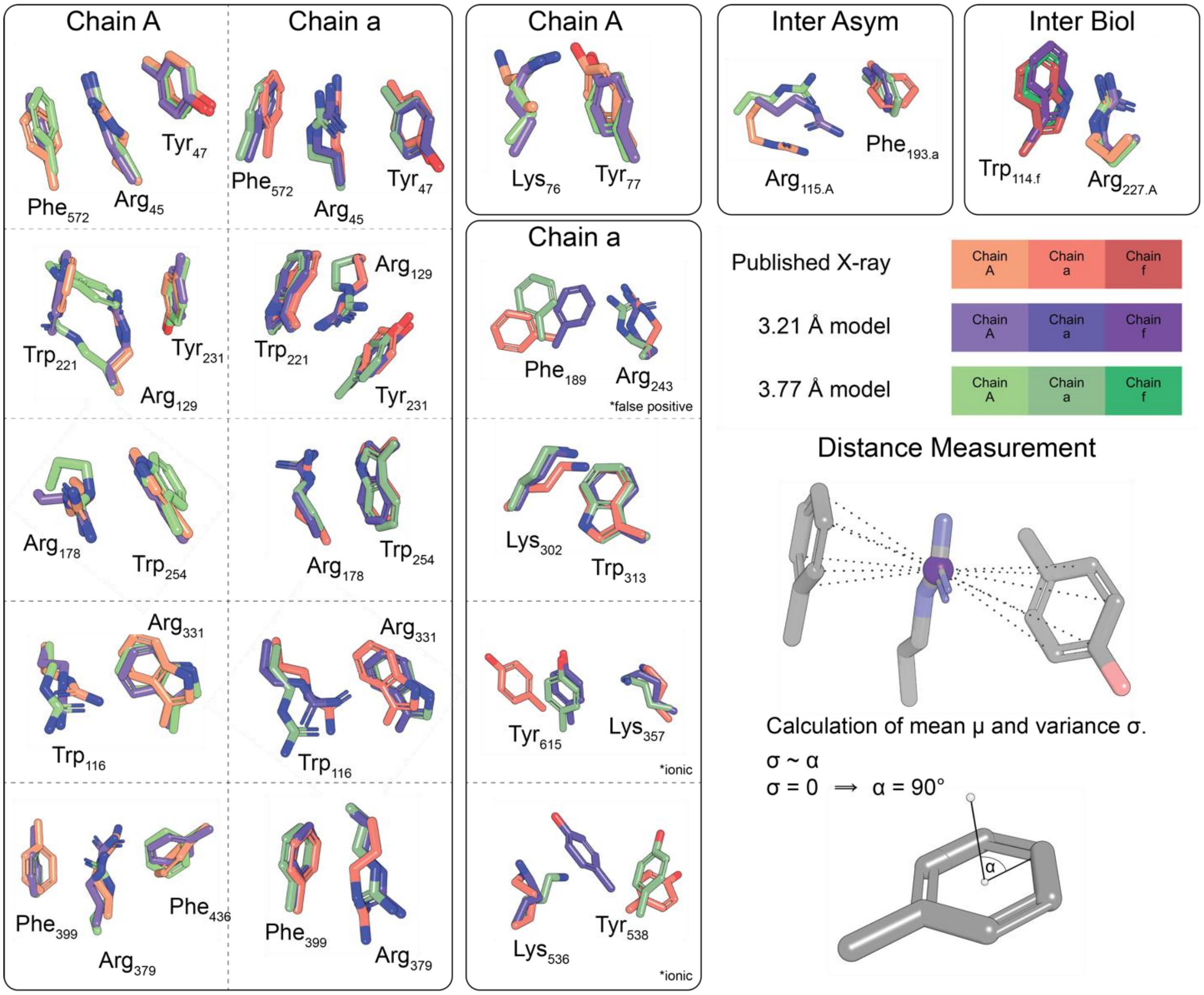

Supplementary Figure 7. Comparison of identified cation-π interactions in the X-ray structure and the cryo-EM structures of 3.77 and 3.2 Å. Residues involved in potential cation-π interactions are shown in stick representation and overlayed utilizing all-atom RMSD (Å). Although the structures from cryo-EM show consistency in recovering cation-π interactions, the X-ray structure does not consistently agree with the cryo-EM determined residue-residue interactions. *Inter Asym* indicates interaction within the two protomers in the asymmetric unit, whereas *Inter Biol* indicates interactions within the biological assembly. On the bottom right, the methodology used for distance measurement to identify cation-π interactions is illustrated: all distances from the cationic moiety to the carbon atoms of the aromatic ring are considered. The variance relates to the angle of the cation concerning the π plane; a variance of 0 indicates a perfect right angle. Details of each cation-π pair can be found in **Supplementary Table 2**.

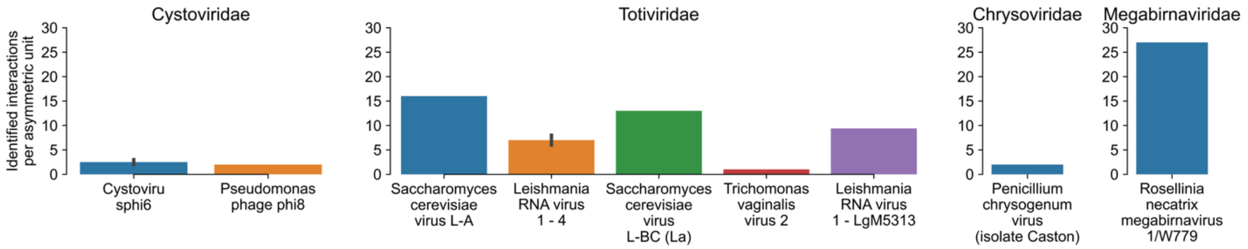

Supplementary Figure 8. Identification of cation-π interactions in *Duplornaviridae*. Occurrence of potential cation-π interactions was analyzed by considering biological assemblies deposited in the protein data bank. Their corresponding taxonomy was also extracted from their PDB annotation. Relative interactions were derived by dividing the total number of identified cation-π pairs by the number of monomeric units reported in each biological assembly of the analyzed PDB entry. Structures considered: 4btg and 5fj5 (Cystoviru sphi6), 4bx4 (Pseudomonas phage phi8), 1m1c (Saccharomyces cerevisiae virus L-A), 6y83 and 7z90 (Leishmania RNA virus 1 - 4), 7qwx (Saccharomyces cerevisiae virus L-BC (La)), 7m12 (Trichomonas vaginalis virus 2), 7ns2 (Leishmania RNA virus 1 - LgM5313), 3j3i (Penicillium chrysogenum virus (isolate Caston)), 8b4z (Rosellinia necatrix megabirnavirus 1/W779). Error bars indicate standard deviation, if more than a single viral capsid structure was available.

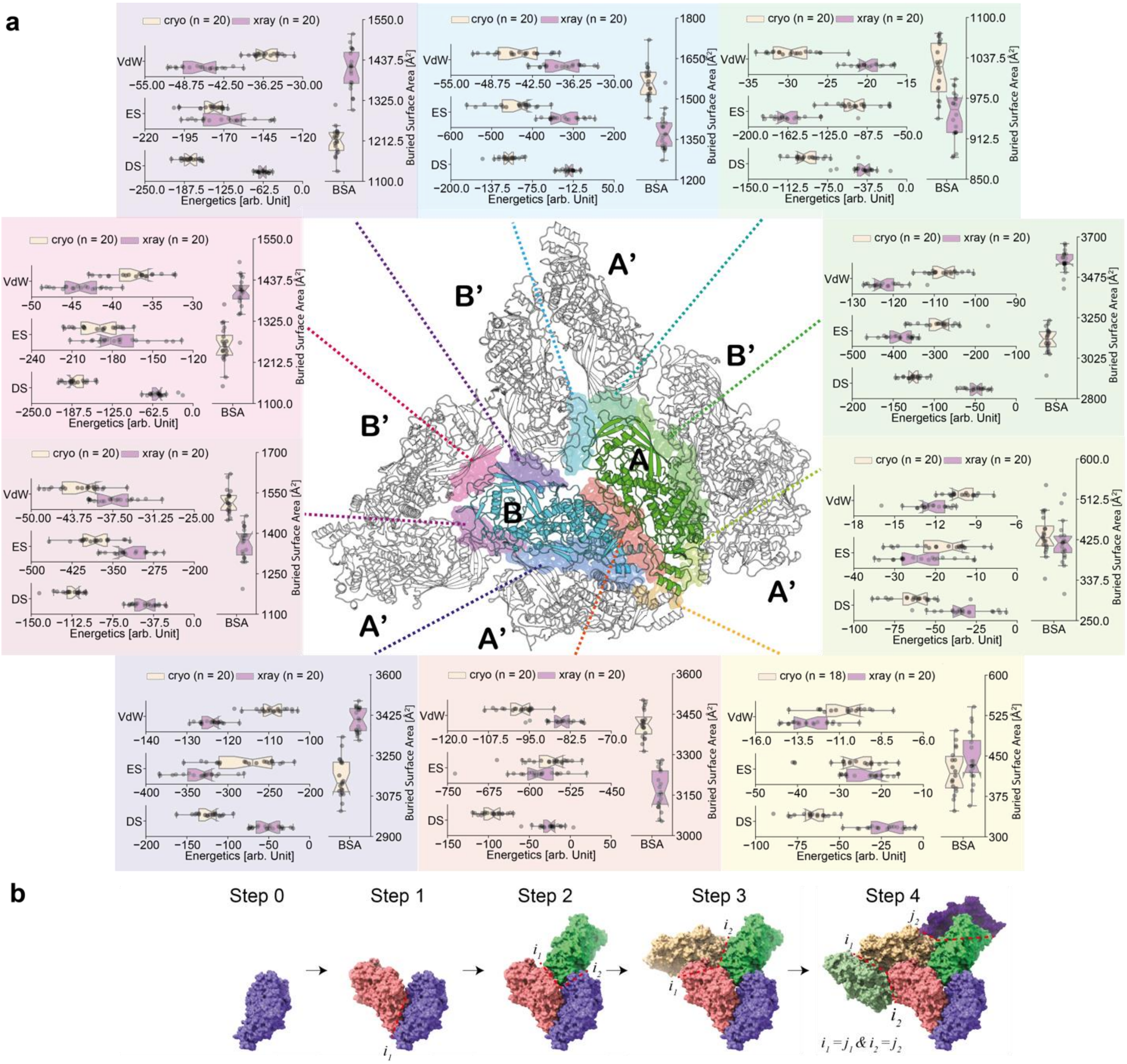

$$E_{\text{total}} = \sum_{i=1}^{n} (E_{\text{vdw}}(i) + E_{\text{es}}(i) + E_{\text{ds}}(i))$$

Supplementary Fig. 9 Comparison of interface energetics forming the crystallographically-resolved and cryo-EM resolved L-A virus capsid. (a) Shown in green (chain A) and blue (chain B) in comic representation is the yeast L-A virus capsomer with adjacent protomers. Subunits corresponding to chain A are labelled with A' and subunits corresponding to B are labelled B'. The colors for the calculated interfaces match the box-plot background color. Calculated were van der Waals forces (VdW), desolvation scores (DE), electrostatics scores (ES) in arbitrary units (a.u.), and the buried surface area (BSA) in Å^2. The box minima represent the 25th percentile, the box maxima the 75th percentile, the Notch indicated the data's median, whiskers extend to the minimum and maximum value inside of a 1.5 interquartile range. All data points are overlayed as a scatter plot. A total number of n=20 models were refined for each condition and interface. (b) Proposed stability analysis (from monomer until step 4) of the capsid informed by the energetics calculations for derived interfaces.

130

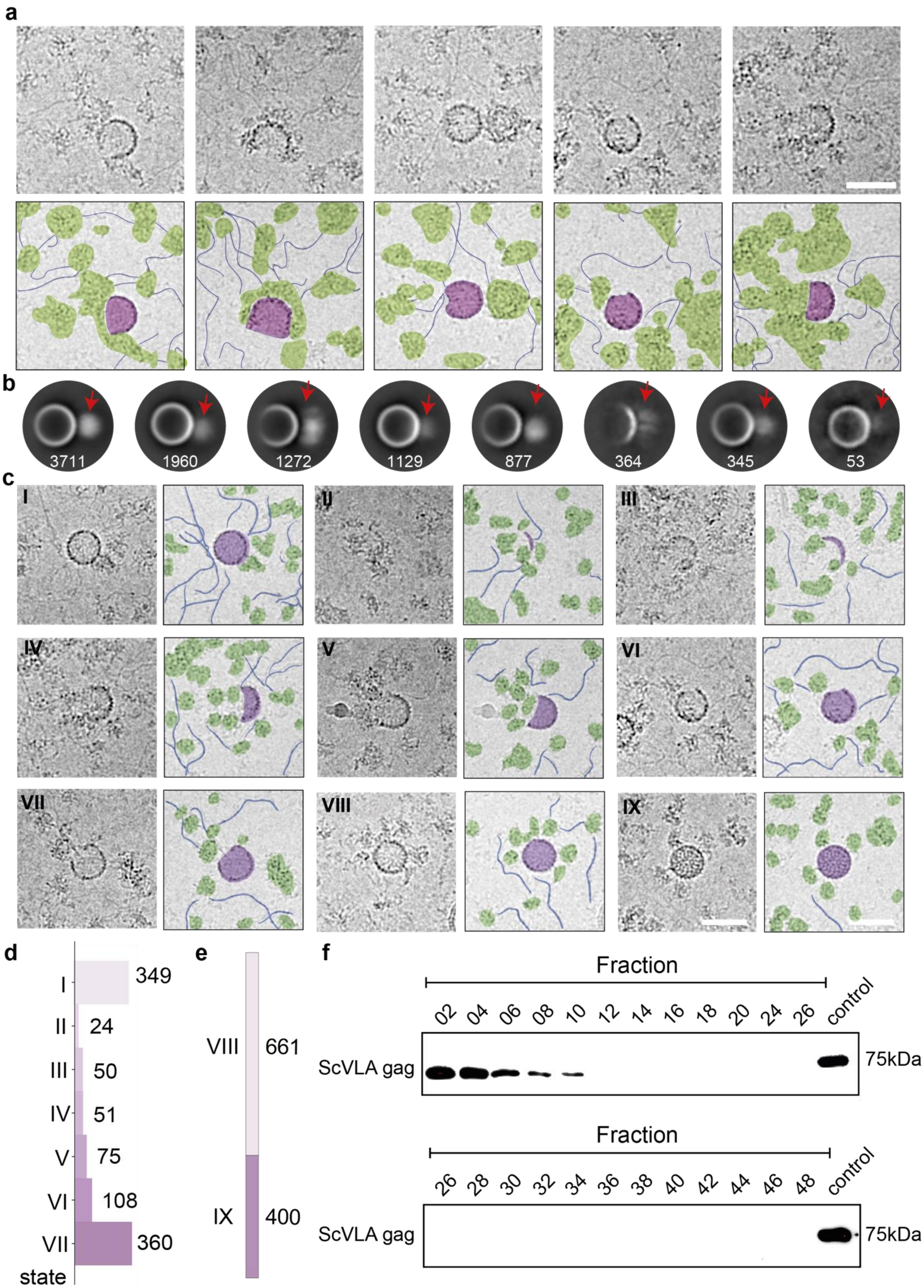
a
b
3711
1960
1272
1129
877
364
345
53
c
I
II
III
IV
V
VI
VII
VIII
IX
d
I
349
II
24
III
50
IV
51
V
75
VI
108
VII
360
state
e
VIII
661
IX
400
f
Fraction
02 04 06 08 10 12 14 16 18 20 24 26 control
ScVLA gag
75kDa
Fraction
26 28 30 32 34 36 38 40 42 44 46 48 control
ScVLA gag
75kDa

Supplementary Fig. 10 L-A virus environment and assembly: (a) A representation of assembly states corresponding to the viral lifecycle is shown in crops and impressions in distinct steps from I to IX, like **Fig. 1a**. The scale bar is 50 nm. (b) 2D classes of the LA virus capsid with diffused densities in proximity; Ribosomes are often bound as shown in, *e.g.*, panel (a). (d) A statistical representation of the assembly states I to VII in absolute numbers. (e) Statistical comparison of full and empty viruses. This shows the ratio between randomly counted full (*N=400*) or empty (*N=661*) mature capsids corresponding to states VIII and IX of (c). (f) Western blot analysis against gag shows signal only in high molecular weight fractions indicating that the virus capsids are not likely to be severely damaged. Statistics of (d) and (e) are derived from manual picking, due to lower contrast of partially assembled particles.

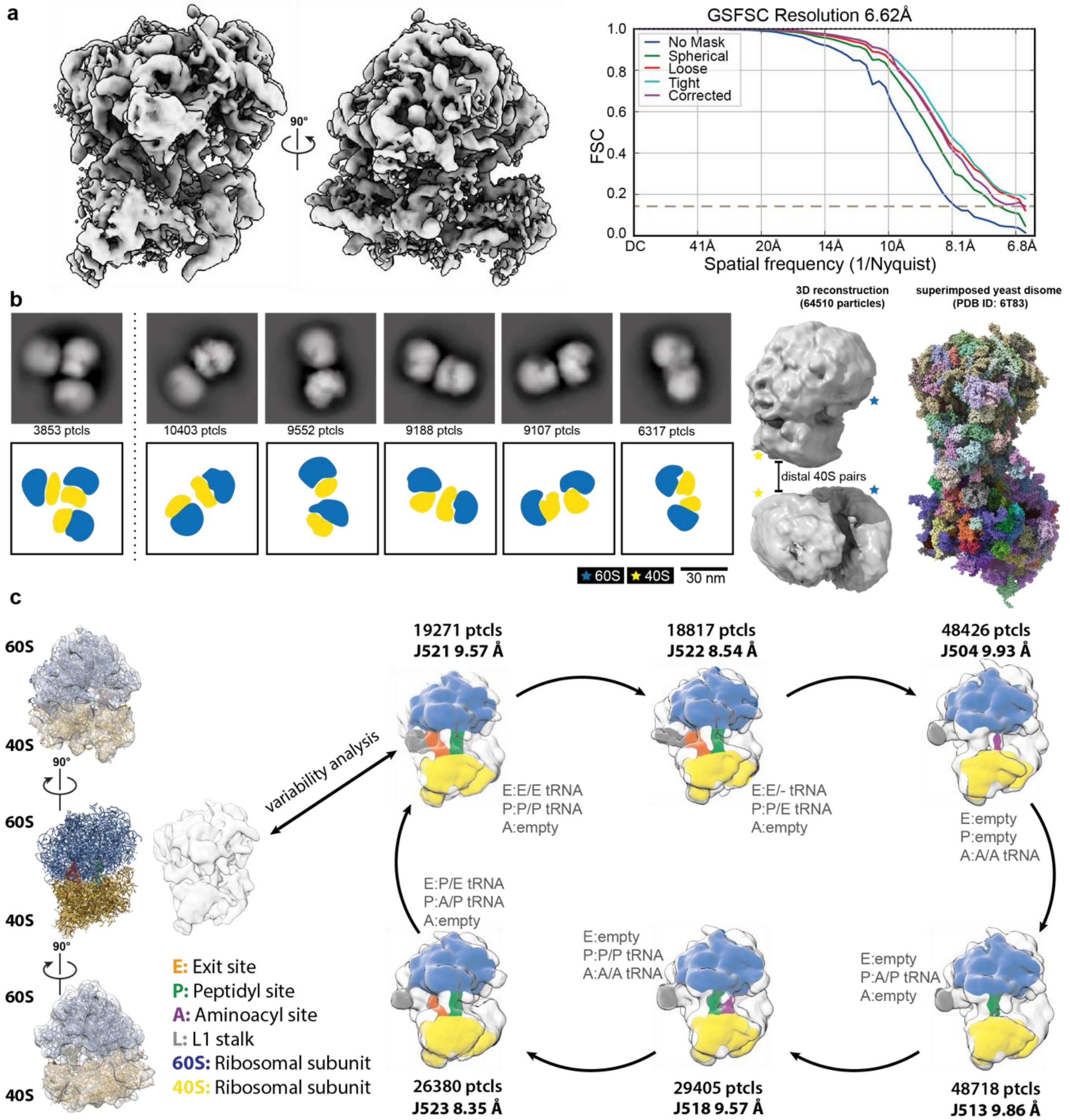

Supplementary Fig. 11 Cryo-EM reconstructions of translationally active ribosomes: (a) 3D reconstructed ribosome at 6.62 Å with an FSC=0.143. (b) 2D class averages and 3D reconstruction of identified polysomes within the native cell extract. Scale bar represents 30 nm. (c) Identified distinct translational states after variability analysis performed in CRYOSPARC.

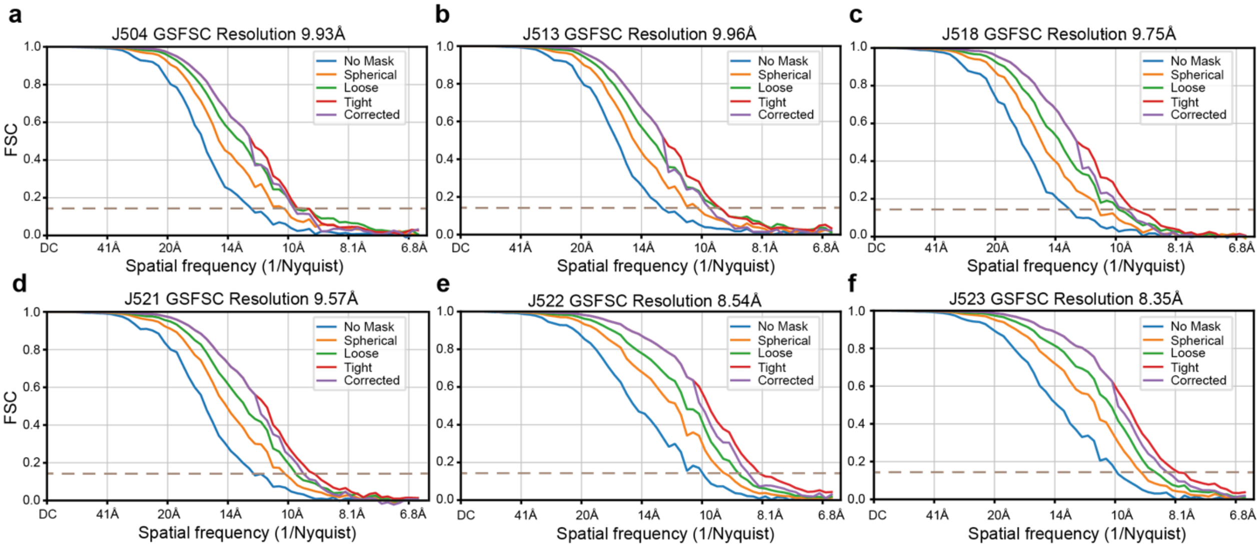

Supplementary Fig. 12 (a-f) FSCs of the 6 distinct translational stages of reconstructed ribosomes after variability analysis shown in **Supplementary Fig. 11c**.

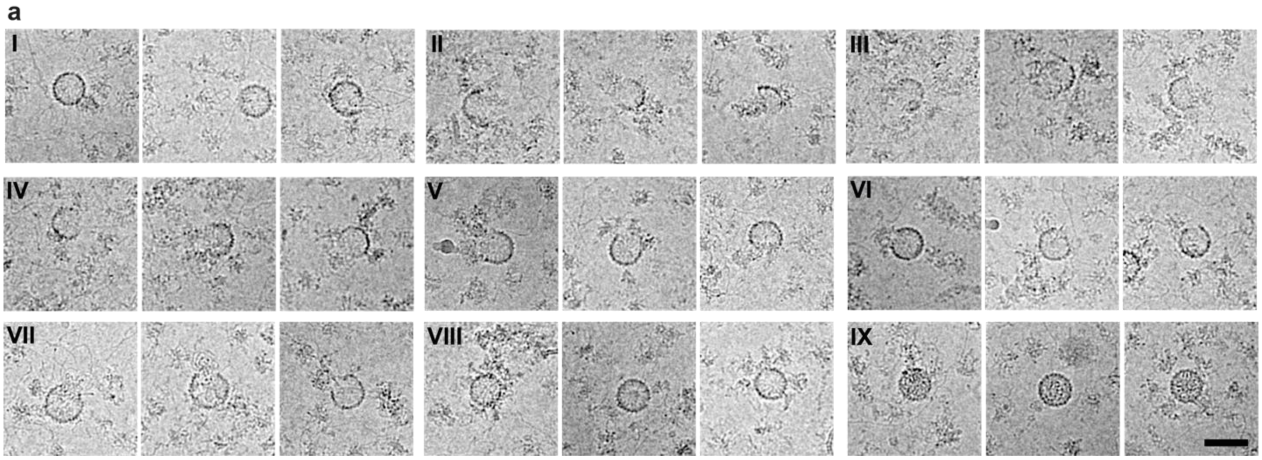

Supplementary Fig. 13 Potential viral life cycle states: (a) More representations of the different states of the viral lifecycle shown in crops, distinctive steps from I to IX. The scale bar represents 50 nm.

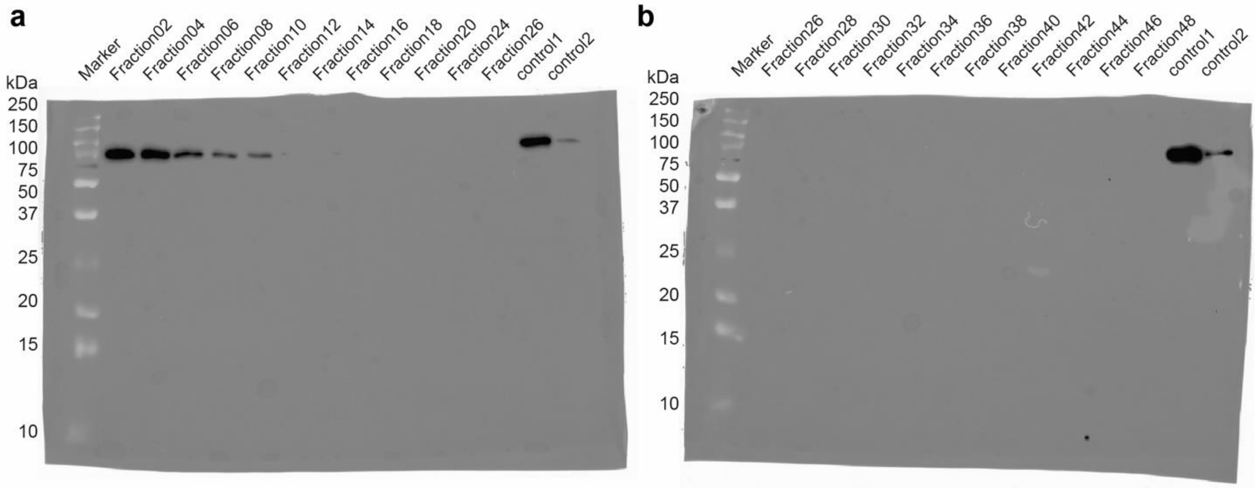

Supplementary Fig. 14 Tracking of the abundance of the L-A helper virus via Western blot analysis: (a, b) Original uncropped membranes after Western blot analysis. Every second fraction was applied on the gel and L-A virus capsid was detected. Control 1 and 2 are positive controls and correspond to the fraction in which the LA virus was detected by mass spectrometry (1), and the yeast lysate used for injecting the SEC (2).

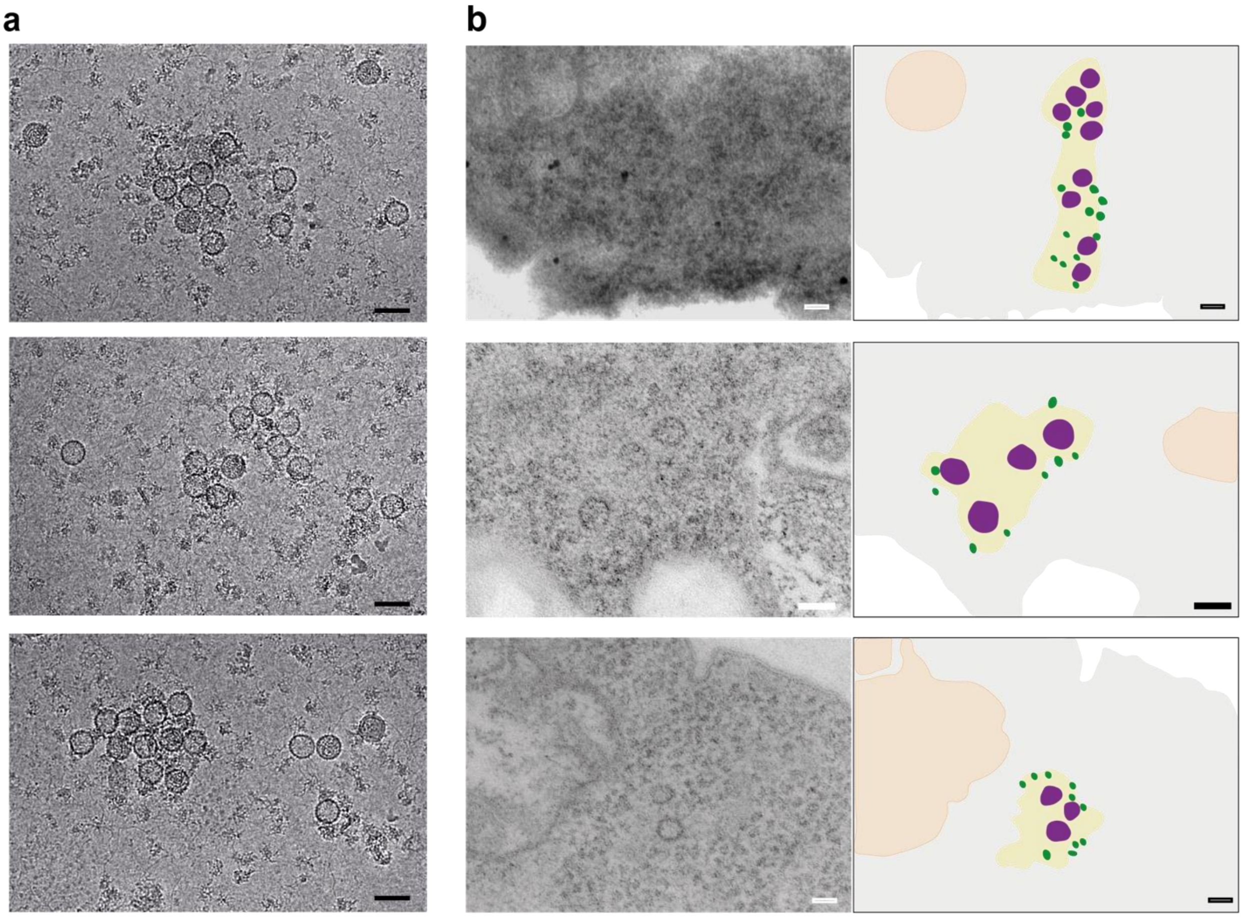

Supplementary Fig. 15 L-A virus communities: (a) More examples of viral particles grouping in the micrographs. Cryo-substituted, in resin embedded *Saccharomyces cerevisiae* sections of 30 nm in width and imaged on the EM 900. Shown are crops through yeast on the left and a comic representation on the right. Vesicles are colored in orange, ribosomes/adjacent proteins dark green, and viral particles in purple (b). Scale bar representing 50 nm.

Supplementary Table 1: Raw results of the CatPi-Toolkit.

cation_resn	cation_resi	cation_chain	pi_resn	pi_resi	pi_chain	mean_dist	std_dist	n	validated	info	uniqueness	type
ARG	45	A	PHE	572	A	4	0.512	6	TRUE		both	intra
ARG	45	A	TYR	47	A	4	0.606	6	TRUE	duplicate	both	intra
LYS	76	A	TYR	77	A	4	0.556	6	TRUE	ionic	unique	intra
ARG	115	A	PHE	193	a	5	0.252	6	TRUE		unique	inter_asym
ARG	129	A	TRP	221	A	4	0.243	6	TRUE		both	intra
ARG	129	A	TYR	231	A	4	0.171	6	TRUE	duplicate	both	intra
ARG	178	A	TRP	254	A	4	0.631	6	TRUE		both	intra
ARG	277	A	TRP	114	f	4	0.463	6	TRUE		unique	inter_assembly
ARG	379	A	PHE	399	A	5	0.375	6	TRUE		unique	intra
ARG	277	E	TRP	114	a	4	0.462	6	TRUE		unique	inter_assembly
ARG	45	a	PHE	572	a	4	0.435	6	TRUE		both	intra
ARG	45	a	TYR	47	a	4	0.65	6	TRUE	duplicate	both	intra
ARG	129	a	TRP	221	a	4	0.285	6	TRUE		both	intra
ARG	129	a	TYR	231	a	4	0.126	6	TRUE	duplicate	both	intra
ARG	178	a	TRP	254	a	5	0.642	6	TRUE		both	intra
ARG	243	a	PHE	189	a	5	0.672	6	FALSE		unique	intra
LYS	302	a	TRP	313	a	4	0.487	6	TRUE		unique	intra
LYS	357	a	TYR	615	a	4	0.641	6	TRUE	ionic	unique	intra
LYS	536	a	TYR	538	a	4	0.571	6	TRUE	ionic	unique	intra
