# Supplementary material for: Delineating organizational principles of the endogenous L-A virus by cryo-EM and computational analysis of native cell extracts
Source: Commun Biol. 2024 May 10;7:557. doi: 10.1038/s42003-024-06204-7 (PMC11087493; doi:10.1038/s42003-024-06204-7)
Supplement: Supplementary file 3 — Description of Additional Supplementary Files [file 42003_2024_6204_MOESM3_ESM.pdf]

Description of Additional Supplementary Files

File name: Supplementary Data
Description: Numerical source data for graphs and charts in the manuscript.

File name: Supplementary Movie 1
Description: Rigid-body assembly of the L-A capsid structure.
